# Temporal concentration and phase synchronization in phase-amplitude coupling

**DOI:** 10.3389/fnbeh.2025.1615997

**Published:** 2025-10-08

**Authors:** Marjan Nosouhi, Stefan Treue, Moein Esghaei

**Affiliations:** ^1^School of Electrical and Computer Engineering, University of Tehran, Tehran, Iran; ^2^Cognitive Neuroscience Laboratory, German Primate Center - Leibniz Institute for Primate Research, Göttingen, Germany; ^3^Faculty of Biology and Psychology, University of Göttingen, Göttingen, Germany; ^4^School of Cognitive Sciences, Institute for Research in Fundamental Sciences (IPM), Tehran, Iran; ^5^Faculty of Computer Science and Engineering, Shahid Beheshti University, Tehran, Iran

**Keywords:** neural oscillations, phase-amplitude coupling (PAC), neural synchrony, temporal concentration, phase synchronization

## 1 Introduction

Neural oscillations are rhythmic patterns of electrical activity generated by groups of neurons. These oscillations occur at different frequencies, ranging from slow rhythms, such as theta (4–8 Hz) to faster rhythms, like gamma (30–200 Hz). Phase-Amplitude Coupling (PAC) is a fundamental feature of brain activity that reflects the interaction between neural oscillations of different frequencies. PAC specifically refers to the phenomenon where the amplitude of high-frequency neural oscillations is modulated by the instantaneous phase of lower-frequency oscillations. This interaction is thought to play a crucial role in coordinating activity across brain regions and controlling information processing (Colgin et al., 2009; Khamechian et al., 2024).

Previous research suggests that PAC is essential for organizing information in the brain and has been linked to cognitive functions such as memory formation, attention, and perception. For instance, theta-gamma PAC has been shown to support memory tasks by synchronizing activity between memory-related regions (hippocampus) and executive control areas (prefrontal cortex) (Canolty and Knight, 2010; Adriano et al., 2010). Similarly, PAC plays a role in attention by helping filter relevant information from distractions.

The study of PAC has also opened new avenues for understanding brain disorders. Abnormal PAC patterns have been observed in patients suffering from epilepsy, Parkinson's disease, and schizophrenia, suggesting that maintenance of PAC is crucial for healthy cognitive and behavioral function (de Hemptinne et al., 2013; Voytek et al., 2015). More recent studies also emphasize the importance of PAC in motor and cognitive dynamics. For example, (Farokhniaee et al. 2023, 2024) demonstrated gait-related and stimulation-induced beta-gamma PAC in the subthalamic nucleus of Parkinson's patients, suggesting PAC as a biomarker for pathological and therapeutic states.

## 2 The PAC paradox: suppressed coupling in working memory

A recent study by Daume et al. measured PAC in the human hippocampus while subjects performed a working memory task (Daume et al., 2024). The study manipulated memory load by varying the number of items participants needed to retain. Contrary to the expectation that PAC would increase with higher cognitive demands, the authors found that PAC was suppressed at higher memory loads. This challenges the conventional view that PAC is essential for inter-regional communication, particularly under conditions requiring high cognitive control (Soheila and Sylvain, 2017). If PAC were a critical mechanism for sustaining working memory under increasing demand, one would expect it to strengthen rather than weaken with greater memory load.

To further explore this paradox, Daume et al. analyzed “noise correlation,” the trial-by-trial correlation of spike rates between neurons. Their analysis focused on neurons whose firing rates correlated with local PAC levels. Interestingly, these neurons exhibited noise correlations that enhanced the information capacity of the local neuronal population, improving working memory representations. This suggests that PAC may still facilitate memory function, despite its suppression under high cognitive load. However, this finding appears contradictory: why would PAC decrease if it plays a crucial role in organizing neural information?

## 3 A proposed framework for untangling PAC mechanisms

Traditional PAC metrics (Adriano et al., 2010; Hülsemann et al., 2019) typically treat PAC as a unitary phenomenon. In previous work (Esghaei et al., 2022), we proposed two distinct mechanisms underlying PAC: temporal concentration and phase synchronization.

**Figure 1:**
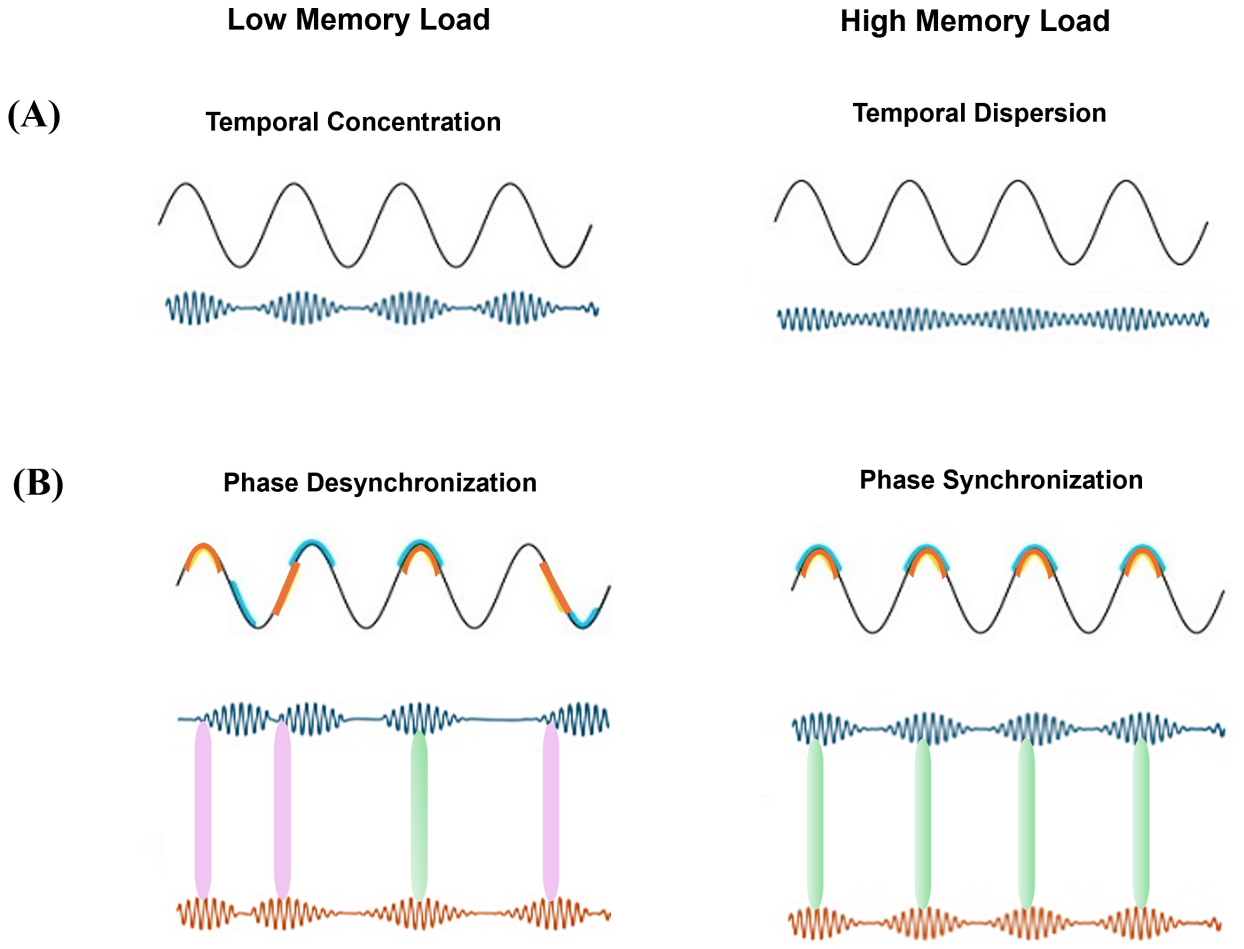
Mechanisms underlying PAC under different memory loads. **(A)** Temporal concentration of gamma bursts. **(Left)** Gamma bursts are tightly concentrated at a specific phase range of the theta cycle (high temporal concentration). **(Right)** Gamma bursts are more temporally dispersed (low concentrated) across phases of a given cycle. **(B)** Phase synchronization. **(Left)** Desynchronized gamma bursts occurring locked to different theta phases, when compared between cycles. This is reminiscent with less co-occurrence of gamma bursts between areas, weakening inter-areal gamma synchrony. **(Right)** All gamma bursts within an area are aligned to a common theta phase, leading to the co-occurrence of gamma bursts across regions. Signals of the two sample brain regions and their preferred theta phase are indicated by blue and orange. Green and red double-sided arrows between the two gamma signals depict temporal co-occurrence and mis-alignment of gamma bursts from the two areas, respectively.

• **Temporal concentration** (see Figure 1A) refers to the time-compression of brief, transient episodes of heightened gamma-band activity (known as gamma bursts). It is noteworthy that these brief oscillatory bursts (reflective of increased local neuronal synchrony), often becomes more compressed into the narrow time windows of certain phases of low-frequency (e.g., theta) oscillations. It is important to note that this compression is independent of the gamma intensity (power), i.e., a gamma burst at a given power may be compressed into a narrower part of the low-frequency cycle (strong temporal concentration) or conversely, distribute wider in time (weak temporal concentration). Strong temporal concentration enhances local network coherence, synchronizing the firing of single neurons within a brain region. In contrast, when gamma bursts are temporally dispersed (i.e., low temporal concentration), neuronal activity becomes less synchronized, which may enhance information coding capacity by reducing redundancy and allowing for more distinct neural representations (Averbeck et al., 2006; Babadi and Sompolinsky, 2014; Esghaei et al., 2018).

• **Phase synchronization** (see Figure 1B) describes the alignment of gamma bursts across brain regions to a particular phase of a common low-frequency oscillation (e.g., theta). This mechanism facilitates inter-regional communication by ensuring that high-frequency activity in different brain areas occurs during optimal excitability windows. Desynchronized gamma bursts across regions weaken inter-areal coordination, potentially disrupting information transfer.

Differentiating between these two distinct mechanisms is crucial for interpreting PAC findings. Conceptually, the first mechanism captures how narrow each given gamma burst occurs in time; while the second mechanism targets the equality of the preferred low frequency phase (where gamma preferably occurs at) across low frequency cycles. Temporal concentration enhances local network coherence but does not necessarily facilitate long-range communication between brain regions. In contrast, phase synchronization aligns neural oscillations across regions, optimizing long-range information transfer. This distinction has been shown promising when distinguishing the contrasting effects of selective attention on PAC. We reviewed previously how attention differentially modulates PAC, in some areas/frequency domains enhancing and in the other suppressing PAC (Esghaei et al., 2022). While these two are theoretically independent, it remains to be examined if they are empirically also independent or not.

## 4 Mechanistic role of PAC at high memory load

In this context, we argue that the findings by Daume et al. primarily reflect changes in temporal concentration, not phase synchronization. The observed reduction in PAC at higher memory loads likely signals decreased temporal clustering of gamma bursts. This decrease could promote a more desynchronized firing pattern, improving information differentiation among neurons.

Thus, rather than signaling a breakdown in hippocampal-prefrontal communication, the reduction in PAC may reflect an adaptive strategy—optimizing memory processing by shifting toward a more flexible and differentiated local neural code. It is important to note that Daume et al. did not specifically quantify whether their observed PAC change was due to temporal de-concentration or phase de-synchronization.

## 5 Discussion

The findings of Daume et al. raise important questions about PAC's role in cognition. If PAC is essential for inter-regional communication, why does it decrease under high cognitive load? Rather than reflecting impaired communication, this reduction may indicate a shift in neural coding strategies. Specifically, PAC neurons may enhance working memory fidelity by introducing noise correlations that improve information encoding at the local population level. At higher memory loads, the brain may optimize memory representations by restructuring how information is maintained.

Many studies do not distinguish between the different PAC mechanisms, making it difficult to compare results or draw clear conclusions. While PAC metrics inherently reflect aspects of phase locking, they do not fully disentangle the distinct contributions of local temporal concentration and inter-regional phase synchronization. PAC can be modulated by changes in either domain, yet these underlying mechanisms have different functional implications. Given the potential confounds introduced by noise, trial selection, and preprocessing variability, we advocate for the use of separate and dedicated measures, such as the Phase-Locking Value (PLV) for assessing inter-areal phase synchronization, and the temporal clustering index (Nosouhi et al., 2025) to quantify local gamma burst alignment within specific low-frequency phase windows. Incorporating these complementary metrics alongside PAC provides a more analytical view of neural coordination, allowing researchers to distinguish whether observed PAC changes arise from alterations in local timing precision or from large-scale communication dynamics and whether or not the two metrics are correlated or not.

It is important to emphasize that temporal concentration PAC, as described in our framework, presupposes the presence of low-frequency oscillations, providing the phase structure necessary for PAC to emerge. Without such an underlying oscillation, mere temporal concentration of gamma bursts would not result in meaningful cross-frequency coupling. Therefore, future investigations should verify the presence and potential sources of these low-frequency components, possibly via spectral decomposition or source localization analyses.

While Daume et al. provide valuable insights into PAC dynamics during working memory, their interpretation may be limited by a focus on noise correlation and temporal concentration. By considering phase synchronization as a distinct mechanism, we propose an alternative explanation: high cognitive loads may still enhance inter-areal communication through phase synchronization PAC, and not temporal concentration PAC. Future studies should aim to disentangle these processes to fully understand PAC's contribution to cognition.
